# The relationship between parenting stress and preschool children’s social–emotional competence: the chain mediating role of parental reflective functioning and parent–child relationship

**DOI:** 10.3389/fpsyg.2025.1679084

**Published:** 2025-09-23

**Authors:** Sisi Yu, Mingying Liao, Xinyi Liu, Yanyan Li

**Affiliations:** ^1^Faculty of Education, Northeast Normal University, Changchun, China; ^2^College of Teacher Education, Sichuan University of Arts and Science, Dazhou, China; ^3^Faculty of Psychology, Southwest University, Chongqing, China

**Keywords:** preschool children, social–emotional competence, parenting stress, parental reflective functioning, parent–child relationship

## Abstract

**Introduction:**

Given the significance of social-emotional competence for the success and well-being of preschool children and the crucial role of the family environment in children’s upbringing, the purpose of this study is to explore the effect of parenting stress on preschool children’s social-emotional competence, and to examine the mediating roles of parental reflective functioning and the parent-child relationship through a chain mediation model.

**Methods:**

A total of 3,166 parents of preschool children aged 3-6 years in China were surveyed using the Parenting Stress Index Short Form, the Chinese Inventory of Children’s Socioemotional Competence, the Child-Parent Relationship Scale, and the Parental Reflective Functioning Questionnaire.

**Results:**

The results showed two main findings: (a) parenting stress, parental reflective functioning, the parent-child relationship, and preschool children’s social-emotional competence were significantly correlated, and parenting stress negatively predicted preschool children’s social-emotional competence; and (b) parental reflective functioning and the parent-child relationship played significant mediating roles between parenting stress and preschool children’s social-emotional competence. The analysis revealed three mediating pathways: (a) the separate mediating role of parental reflective functioning; (b) the separate mediating role of the parent-child relationship; and (c) the chain mediating role of parental reflective functioning and the parent-child relationship.

**Discussion:**

This study contributes to research in the field of social-emotional competence and provides a theoretical basis for improving preschool children’s social-emotional competence.

## 1 Introduction

Social–emotional competence refers to the synthesis of knowledge, skills and attitudes of people’s social–emotional competence, specifically including four dimensions: cognitive control, affective expression ability, empathy and prosocial behavior, and emotion regulation (Li et al., 2020). In the context of the new technological and scientific revolution, social–emotional competence is widely recognized as one of the essential characteristics that distinguish humans from artificial intelligence, and is regarded as a core competence necessary for talent in the 21st Century (Liu J. et al., 2021; Liu Z. et al., 2021). Social–emotional competence has generally undergone four stages from its emergence to development—social intelligence, multiple intelligence, emotional intelligence, and social–emotional competence (Xu, 2020).

Social–emotional competence is an important skill for children’s growth and development. A host of studies have shown that good social–emotional competence exerts an important influence on children’s classroom adaptation and academic performance (Denham et al., 2014), and that children with higher social–emotional competence show greater ability in the establishment of social relationship and social adaptation (McCabe and Altamura, 2011). On the contrary, a lot of symptoms may be found in children with poor social–emotional competence: difficulty in adaptation, emotional and behavioral problems, difficulty in learning (Bornstein et al., 2010), and possibly, physical and psychological health problems (Im et al., 2019). Studies have shown that social–emotional competence is a kind of teaching skill that can be fostered among preschool children (Fang, 2023). Generally, the preschool period refers to the period of early childhood development typically ranging from 3 to 6 years of age, a critical period for the development of various competencies of preschool children. During the preschool period, children are more sensitive to environmental stimuli which may promote or hinder the development of their social–emotional competence. Some studies have shown that early intervention services will influence children’s development. In other words, children who accept early intervention services show better social–emotional competence (Chavez et al., 2024). Therefore, it is of great significance to foster a supportive and responsive parenting environment for preschool children in the critical preschool period to cultivate their social–emotional competence.

Various factors in the family will inevitably have an influence on children’s development (Hooper et al., 2023). A positive family environment can promote the development of children’s social–emotional competence, while a negative will have a negative influence in this regard (Xu et al., 2024). The economic and energy input for raising a child keeps increasing as social competitions intensify. Most parents are faced with the increasing challenge of raising their children, while bearing heavy burdens from work and daily life (Fang et al., 2024; Liu et al., 2015). Parenting stress refers to a series of stresses caused by the parents’ own characteristics, disordered parent–child relationship, and children’s behaviors when parents fulfill their roles and interact with their children, including three dimensions: parenting distress, parent–child dysfunction interaction, and difficult children (Abidin and Brunner, 1995). Studies have shown that parents in China are under greater stress to raise their children than those in other countries (Li et al., 2017). Parenting stress can not only directly influence children’s emotional symptoms and conducts, but also influence their development through reduced positive parenting behaviors (Hao et al., 2019). It is visible that parenting stress is an important challenge for children’s growth and development, so exploring how parenting stress influences pre-school children’s social–emotional competence is conducive to the timely prevention of social–emotional problems among preschool children.

### 1.1 Parenting stress and preschool children’s social–emotional competence

As stated in the family system theory, the family is deemed as a system comprised of family members who are mutually influenced and linked. In a family, parents have the longest and closest contact with preschool children. Children’s social–emotional competence is initially developed in this microsystem. The Family Stress Model (FSM) posits that stressors undermine caregivers’ emotional and cognitive resources, heighten negative affect and increase within-family conflict, and spill over into harsher, less sensitive, or withdrawn parenting, which in turn constrains children’s self-regulation, empathy, and prosocial behavior (Conger et al., 1992; Conger and Donnellan, 2007). Such patterns limit opportunities for emotion coaching and collaborative problem-solving, which are foundational to preschoolers’ social–emotional competence (Nievar et al., 2014; Masarik and Conger, 2017). The Conservation of Resources (COR) theory conceptualizes stress as actual or threatened loss of key resources and predicts compensatory behaviors aimed at conserving remaining resources (Hobfoll, 1989; Hobfoll et al., 2018). Under sustained parenting stress, parents are more likely to adopt efficiency-oriented, controlling interaction strategies and to reduce emotionally rich, mentalization-laden dialogue. This curtails children’s opportunities for emotion socialization and co-regulation, impeding the development of emotion understanding, conflict resolution, and peer adaptation—core facets of social–emotional competence. Thus, it can be inferred that higher levels of parenting stress may be associated with lower levels of social–emotional competence, with this association mediated by the availability of parental emotional and behavioral support from parents.

Parenting stress has been proven to be closely related to children’s emotions and behaviors (Cucinella et al., 2022; Tan et al., 2012). A positive and harmonious family environment fosters a safer and more comfortable surrounding for children, which is conducive to the healthy development of their emotions. On the contrary, children will be immersed in anxiety and tension amid a negative and conflicting family environment, leading to emotional and behavioral problem. Parenting stress is a kind of negative emotional state in the family environment, which not only influences parents themselves, but also influences the development of children together with other factors. Greater parenting stress brings a higher probability of problematic behaviors among children (Gao et al., 2020). Possible explicit behavioral problems of preschool children can be directly and significantly predicted based on mothers’ parenting stress (Jia et al., 2024). Fathers’ parenting styles have a mediation effect on the relationship between fathers’ parenting stress and children’s implicit and explicit problematic behaviors. Therefore, fathers’ parenting styles soften the negative influence of parenting stress on children’s behaviors to a certain extent (Liu et al., 2015). Some researchers explored the relationship between family environment, parenting stress and children’s emotional intelligence, finding out that parenting stress may exert a negative influence on children’s emotional intelligence by reducing parents’ positive parenting behaviors and increasing negative ones (Feng and Wang, 2023).

Parenting stress is closely correlated to the development of children’s social competence (Guajardo et al., 2010). The socialization theory emphasizes parents’ key role in children’s development. While facilitating children’s academic achievements, parents can also promote the development of children’s social competence by offering educational resources and related support (Li, 2015). When feeling less parenting stress, parents have greater parenting self-efficacy. Parents with greater parenting self-efficacy may better cope with stresses and challenges during parenting and reduce children’s anxiety and depression on one hand and are more likely to engage in positive and supportive parenting, e.g., encouragement, praise and proper discipline, on the other hand. All these behaviors are conducive to the development of children’s social–emotional competence, as well as the attainment of their academic achievements (Pan et al., 2025). Some studies, exploring the relationship between grandparents’ co-parenting and children’s social competence, found out that mothers’ parenting stress and their parenting self-efficacy have chained mediation effects in the relationship between grandparents’ co-parenting and children’s social competence. Specifically speaking, grandparents’ coparenting helps mothers relieve their parenting stress, enhance their parenting self-efficacy, and ultimately promote the development of children’s social competence (Gong et al., 2021).

To sum up, the current research on parenting stress and preschool children’s development mainly focuses on children’s emotions, behavioral problems and social competence. There is little research on the relationship between parenting stress and preschool children’s social–emotional competence. Nevertheless, existing literature shows that negative parenting stress among parents will exert a negative influence on preschool children’s social–emotional competence. Preceding paragraphs also suggest that cultivating preschool children’s social–emotional competence plays a vital role in preschool children’s physical and psychological health, academic achievements, and social adaptability. Therefore, this study explores the relationship between parenting stress and preschool children’s social–emotional competence, as well as the corresponding action mechanism to systematically uncover the influence mechanism of preschool children’s social–emotional competence, thus relieving parenting stress and providing support and reference for the improvement of preschool children’s social–emotional competence.

### 1.2 Mediation effect of parental reflective functioning and parent–child relationship

This study further explores the mediation effect of parental reflective functioning and parent–child relationship in parenting stress and preschool children’s social–emotional competence based on the mentalization theory and attachment theory. Parental reflective functioning refers that parents can understand and explain children’s behaviors based on children’s psychological states (Ordway et al., 2015). It is a stable protective factor. On the one hand, parental reflective functioning can mitigate the negative influence of parents on preschool children’s development. For example, Dollberg et al. (2021) found out that mothers’ reflective function can be used as an important factor to buffer the negative influence of parents’ general anxiety on children. On the other hand, parental reflective functioning can also ease parents’ problems. For example, Shalev et al. (2023) found out that promoting parental reflective functioning can reduce the burden of guilt on parents when exploring the relationship between parents’ guilt and children’s problems. In addition, parental reflective functioning can also mitigate the influence of children’s negative factors on their development. For example, Wong et al. (2017) found out that parental reflective functioning can serve as a protective factor to mitigate the potential adverse influence of children’s negative emotions on their behavioral problems in the presence of other risk factors related to early behavioral problems. Other studies have found that parenting stress can be used to predict parental reflective functioning. Greater parenting stress results in lower scores of certainties, interest and curiosity in parental reflective functioning. However, reduction of pre-mentalization can help parents relieve their parenting stress and improve the psychological health of themselves and their children (Nijssens et al., 2018). Therefore, parental reflective functioning may have a mediation effect on parenting stress and preschool children’s emotional competence.

According to the family system theory, all members of the family system are inter-actional and mutually influenced. Being a part of the family system, parents have more negative emotions and experiences under parenting stress, which can be communicated to children during interaction among family members. In the family system, the factors influencing children’s development include near-end and far-end ones. Far-end factors include parenting stress, family structure, etc., while near-end factors cover parenting behaviors, parent–child relationships, etc. The former can influence children’s development directly or indirectly through a near-end factor. According to the susceptibility-stress-adaptation model, greater parenting stress of mothers will indirectly influence children’s development outcomes through such family environment characteristics as parent–child relationship and husband-wife relationship (Wang et al., 2020). The safety attachment theory provides a theoretical framework for describing and explaining the emotional connection between children and their main caregivers. Interaction relationship refers to the interpersonal relationship fostered during the interaction between children and their parents in daily life based on blood relation or common life, including parent–child intimacy and parent–child conflict (Driscoll and Pianta, 2011). Studies have shown that parent–child relationships can help children foster positive and stable emotions (Cassidy, 1994). Therefore, a good parent–child relationship empowers better development of children’s social–emotional competence. On this basis, this study takes the parent–child relationship as the second mediating variable to explore its mediation effect on parenting stress and preschool children’s social–emotional competence.

Previous studies have shown that parental reflective functioning is a robust predictor of the quality of the parent–child relationship, and that parents’ ability to reflect on their children’s psychological state during parent–child interactions may be key to improving parent–child relationships (Rostad and Whitaker, 2016). Stover and Kiselica (2014) found out that higher reflection in mothers has a positive impact on parenting, as such parents can better understand their children’s behaviors and meet their needs, resulting in a stronger parent–child bond. By exploring the relationship between childhood mal-treatment experience, parental reflective functioning, and the parent–child relationship, Liu J. et al. (2021) and Liu Z. et al. (2021) discovered that enhancing parental reflective functioning is essential for improving the parent–child relationship and facilitating children’s healthy development. Therefore, this study assumes that parental reflection functioning and the parent–child relationship has a mediated effect on parenting stress and preschool children’s social and emotional competence.

### 1.3 The present study

In summary, although previous studies have explored the relationships between parenting stress, parental reflective functioning, parent–child relationship, and children’s social–emotional competence from various perspectives, several limitations remain. First, while extensive research has documented the predictors and outcomes of social–emotional competence in school-aged, investigations focusing on the foundational preschool period remain disproportionately scarce. The unique socio-cognitive characteristics and plasticity of early childhood warrant dedicated investigation. Secondly, although the mediating role of parent–child relationships is well-established, the parental reflective functioning has been understudied as a primary proximal mechanism linking parenting stress to child outcomes. Its potential role as the initial catalyst in the stress transmission pathway remains inadequately empirically validated. Finally, existing studies tend to examine parenting stress, reflective functioning in parents, parent–child relationships, and child social–emotional competence in isolation or in pairs. A paucity of research exists that provides a comprehensive theoretical framework capable of simultaneously examining how these factors interact through mediating pathways to shape children’s developmental outcomes. By examining the chain mediation model, this study attempts to reveal the multi-layered, progressive pathways through which parenting stress affects preschool children’s social–emotional abilities.

This study specifies a chained mediation model in which parental reflective functioning and the parent–child relationship transmit the effect of parenting stress on preschoolers’ social–emotional competence. In comparison with previous studies, the innovation of this study lies in three key aspects. Firstly, it simultaneously incorporates parental reflective functioning and parent–child relationship within the same model to examine their chained effects. Secondly, it focuses on the preschool stage of social–emotional competence’s rapid growth. Thirdly, it statistically controls potential confounders such as children’s gender and family socioeconomic status.

Grounded in the Family Stress Model and Conservation of Resources theory, and informed by mentalization and attachment security perspectives, the following inferences can be drawn. Firstly, Parenting stress depletes parental affective or cognitive resources and increases negative spillover into caregiving, reducing sensitive responsiveness and emotional availability. This constrains children’s emotion socialization and peer adaptation opportunities, lowering social–emotional competence. Secondly, under stressor load, executive and affect regulatory resources are taxed, reducing parental reflective functioning. Lower parental reflective functioning impairs accurate representation and contingent response to children’s mental states, diminishing mentalization-rich dialogue and co-regulation that scaffold social–emotional competence. Thirdly, parenting stress diffuses into the parent–child subsystem, lowering closeness and increasing conflict and inconsistency. This degrades children’s security and the quality of their socioemotional learning context, reducing social–emotional competence. Lastly, parenting stress first compromises parents’ internal mentalization resources—parental reflective functioning (representation layer). Reduced parental reflective functioning manifests as lower sensitivity emotional availability, which cumulates in poorer parent–child relationship (context layer). In this climate, children receive fewer opportunities for mentalization and emotion socialization, constraining social–emotional competence.

Accordingly, the following four hypotheses have been formulated.

Hypothesis 1: Parenting stress is significantly negatively correlated with preschool children's social-emotional competence.

Hypothesis 2: Parental reflective functioning has a mediation effect in the relationship between parenting stress and preschool children's social-emotional competence.

Hypothesis 3: Parent-child relationship has a mediation effect in the relationship between parenting stress and preschool children's social-emotional competence.

Hypothesis 4: Parental reflective functioning and parent-child relationships have a chained mediation effect on the relationship between parenting stress and preschool children's social-emotional competence.

## 2 Methods

### 2.1 Participants and procedure

This study was conducted in Sichuan Province of China. These regions boast a large population base and a diverse demographic profile, potentially mitigating the impact of intercultural differences on our findings and enhancing their representativeness for Chinese preschool children. A total of 3,276 preschoolers’ parents completed the questionnaire. Before distributing the questionnaires and evaluating the children’s performance, the authors communicated with the principals of the kindergartens about the study’s purpose. Upon receiving the kindergartens’ consent, they distributed the questionnaires to parents online via wjx.cn. Parents who wanted to participate were directed to click on the provided link to complete an informed consent form and a formal questionnaire. The questionnaires asked parents to report on their parenting stress, reflective function, and parent–child relationship. At the same time, parents also scored their children’s social–emotional competence (on a scale of 1 to 5) based on their behavior and performance.

After eliminating respondents who did not provide careful responses (as indicated by a response time of less than 25 s), a dataset of 3,166 valid responses was obtained, including 1,534 parents of boys (48.45%) and 1,632 parents of girls. Of the children, 16.17% were 3 years old, 28.11% were 4 years old, 34.68% were 5 years old, and 21.04% were 6 years old. Among the children, 984 (31.08%) were only children, while 2,182 (68.92%) were not. Of the parents surveyed, 728 were fathers (22.99%), and 2,438 were mothers (77.01%). 19.58% of parents were only children, while 80.42% were not. 29.12% of parents were under 30 years old, 40.81% were aged 31–35, and 21.73% were aged 36–40. 8.34% of parents were over 40 years old. 60.96% of parents had a high school education or less; 16.99% had a college education; 21.1% had a bachelor’s degree; and 0.95% had a master’s degree or above. 34.74% of parents had an annual household income of less than 30,000 yuan; 32.79% had an income between 30,000 and 8,000 yuan; 18% had an income between 90,000 and 14,000 yuan; and 8.47% had an income between 15,000 and 20,000 yuan. More than 200,000 yuan accounted for 6%.

### 2.2 Measures

#### 2.2.1 Measurement of parenting stress

This study adopted the Parenting Stress Index Short Form (PSI-SF), which was formulated by Abidin (1990) and revised by Yeh et al. (2001), to measure parenting stress. The form was designed to measure three observations: parenting distress, parent–child dysfunction interaction, and difficult children, each of which contains 12 items, 36 items in total. The Likert 5-point scoring system was adopted, with 1 representing “Strongly Disagree” and 5 representing “Strongly Agree.” The form showed good psychometric characteristics and was well consistent with its three sub-forms internally. In this study, the Cronbach’s Alpha of the form was 0.977.

#### 2.2.2 Measurement of preschool children’s social–emotional competence

This study adopted the Chinese Inventory of Children’s Socioemotional Competence (CICSEC) formulated by Li et al. (2020). The inventory is comprised of cognitive control, affective expression ability, empathy and prosocial behavior, and emotion regulation, with a total of 30 questions offered. Among them, the 1st, 2nd, 3rd, 5th, 6th, 7th, 8th, 9th, 10th, 11th, 12th, 13th, 25th, 26th, 28th, 29th, and 30th questions are reverse scoring questions. The Likert 5-point scoring system was adopted, i.e., Preschool children’s get higher scores show better social–emotional competence. CICSEC showed good psychometric characteristics and was well consistent with its four sub-inventories internally, with 1 representing “Strongly Disagree” and 5 representing “Strongly Agree.” In this study, the Cronbach’s Alpha of CICSEC was 0.866.

#### 2.2.3 Measurement of parent–child relationship

This study adopted the Child–Parent Relationship Scale (CPRS) formulated by Pianta (1992) and revised by Zhang et al. (2008). The scale is comprised of three dimensions: intimacy, dependence and conflict. However, as the dependence enjoys poor reliability and validity in China, this study only gave a total of 22 questions from two dimensions of intimacy and conflict, including 10 questions for intimacy and 12 ones for conflict. This study got the total score of the parent–child relationship by adding reverse scores from the dimension of conflict to scores from the dimension of intimacy. The Likert 5-point scoring system was adopted, with 1 representing “Completely Inconsistent” and 5 representing “Completely Consistent.” The scale showed good psychometric characteristics and was well consistent with its two sub-scales internally. In this study, the Cronbach’s Alpha of the scale was 0.873.

#### 2.2.4 Measurement of parents’ reflective function

This study adopted the Parent Reflective Functioning Questionnaire (PRFQ) formulated by Luyten et al. (2017) and revised by Chen et al. (2022) to measure parental reflective functioning. The questionnaire contains questions about pre-mentalization (5 questions), certainty (6 questions) and interests and curiosity (5 questions), 16 questions in total. Among them, 9 questions are reverse scoring questions. The pre-mentalization is the non-pre-mentalization mode of parents with impaired reflective function, and the higher the score, the more difficult it is for parents to accurately understand and explain their children’s psychological experience. Therefore, this study got the total score of parental reflective functioning by adding reverse scores from the dimension of pre-mentalization to scores from dimensions of certainty and interests and curiosity. The Likert 7-point scoring system was adopted, with 1 representing “Strongly Disagree” and 7 representing “Strongly Agree.” The form showed good psychometric characteristics and was well consistent with its four sub-forms internally. In this study, the Cronbach’s Alpha of the questionnaire was 0.784.

### 2.3 Data analysis

The single factor analysis in SPSS 27.0 was applied to test whether there is a common method bias in the data. In this study, descriptive statistics were adopted to analyze the general situation of parents’ parenting stress, parent’s reflective function, parent–child relationship, preschool children’s social–emotional competence, and the basic situation of each dimension. Different demographic difference test methods were used according to the types of demographic variables to analyze the differences of each variable in demographic variables. Pearson correlation test was applied to explore the correlation between variables. The Process plug-in of SPSS 27.0 was used to test the direct effect of parents’ parenting stress on preschool children’s social–emotional competence, as well as the mediation effect of parents’ reflective function and parent–child relationship between them. Finally, the significance of different paths was tested by applying the Bootstarp method.

## 3 Results

### 3.1 Common method bias test

Since all the data were from parents’ reports, there might be common method bias. Therefore, the common method bias of questionnaire was analyzed by using the Harman single-factor method before data analysis. The results showed that the unrotated first factor only explains 30.76% of the total variances, less than 40% of the total variances, indicating that there is no obvious common method bias in this study.

### 3.2 Preliminary analysis

Firstly, on a scale from 1 to 5, the mean score for preschool children’s social–emotional competence was 3.492 (*SD* = 0.577), which is above the scale’s theoretical midpoint (3.00). There were significant differences by gender (*t* = −4.683, *p* < 0.001), kindergarten location (*t* = −6.366, *p* < 0.001), kindergarten type (*t* = −2.438, *p* < 0.05), age (*F* = 3.179, *p* < 0.05), parents’ education levels (*F* = 9.623, *p* < 0.001), and annual household income (*F* = 12.299, *p* < 0.001). By gender, girls showed higher social–emotional competence than boys; by location of kindergarten, children in urban areas showed higher social–emotional competence than those in rural areas; by nature of kindergarten, children in public kindergartens showed higher social–emotional competence than those in private kindergartens; by age, 5-year-old preschool children showed the best social–emotional competence, while 3-year-old preschool children showed the worst; by parents’ education levels, the parents with high school degree or below showed the best social–emotional competence, while parents with master’s degree or above showed the worst; and by annual household income, the children in families with an annual household income of over RMB 150,000 showed the best social–emotional competence, while the children in families with an annual household income of less than RMB 30,000 showed the worst.

Secondly, on a 1–5 scale, the mean score for parenting stress was 2.303 (*SD* = 0.984), which is below the scale’s theoretical midpoint (3.00). There were significant differences by only-child status (*t* = −4.391, *p* < 0.001), kindergarten location (*t* = 5.721, *p* < 0.001), parents’ age (*F* = 5.918, *p* < 0.001), parents’ education levels (*F* = 11.362, *p* < 0.001), and annual household income (*F* = 13.404, *p* < 0.001). By whether the child is the only child, the parents of the non-only child showed greater parenting stress than those of only child; by location of kindergarten, the parents of children in township kindergartens showed greater parenting stress than those in cities; by parents’ age, the parents aged 36 ~ 40 showed the highest level of parenting stress, while the parents under 30 showed the lowest level; by parents’ education levels, the parents with high school degree or below showed the highest level of parenting stress, while parents with master’s degree or above showed the lowest level; and by annual household income, the level of parenting stress decreases with the increase of annual household income.

Thirdly, on a 1–5 scale, the mean score for the parent–child relationship was 3.675 (*SD* = 0.675), which is above the scale’s theoretical midpoint (3.00). There were significant differences by the child’s only-child status (*t* = 5.894, *p* < 0.001), parents’ only-child status (*t* = 2.147, *p* < 0.05), kindergarten location (*t* = −7.795, *p* < 0.001), age (*F* = 6.253, *p* < 0.001), parents’ education levels (*F* = 11.528, *p* < 0.001), and annual household income (*F* = 15.252, *p* < 0.001). By whether the child is the only child, the only child showed a more harmonious relationship with his / her parents; by whether parents are the only child, the only-child parents showed a more harmonious relationship with their children; by location of kindergarten, the children attending urban kindergartens showed more harmonious relationship with their parents; by age, the 3-year-old children those of harmonious relationship with their parents, while the 6-year-old children showed the worst; by parents’ education levels, the parents with college degree and above showed the most harmonious relationship with their children, while parents with high school degree or below showed the worst; and by annual household income, the parent–child relationship becomes more harmonious with the increase of annual household income.

Finally, on a 1–5 scale, the mean score for parental reflective functioning was 4.734 (*SD* = 0.922), which is above the scale’s theoretical midpoint (3.00). There were significant differences by gender (*t* = −2.283, *p* < 0.05), the child’s only-child status (*t* = 7.495, *p* < 0.001), parents’ only-child status (*t* = 3.964, *p* < 0.001), kindergarten location (*t* = −8.374, *p* < 0.001), age (*F* = 7.712, *p* < 0.001), parents’ education levels (*F* = 20.717, *p* < 0.001), and annual household income (*F* = 22.667, *p* < 0.001). By gender, the parents of girls showed higher reflective function than those of boys; by whether child is the only child, the parents of the only child showed higher reflective function than those of the non-only child; by whether parents are the only child, the only-child parents showed higher reflective function than non-only-child parents; by location of kindergarten, the parents of children attending urban kindergartens showed higher reflective function than those of children attending township kindergartens; by age of children, the parents of 6-year-old children showed the highest level of reflective function, while parents of 3-year-old children showed the lowest; by parents’ education levels, parents with college degree and above showed the highest level of reflective function, while parents with high school degree or below showed the lowest; and by annual household income, the level of parental reflective functioning gradually increases with the increase of annual household income.

### 3.3 Correlation analysis

The correlation analysis of four variables (as shown in Table 1) showed that preschool children’s social–emotional competence is significantly negatively correlated with parenting stress (correlation coefficient of −0.586), but significantly positively correlated with parent–child relationship (correlation coefficient of 0.659) and parental reflective functioning (correlation coefficient of 0.445); the parenting stress is significantly negatively correlated with parent–child relationship (correlation coefficient of −0.644) and parental reflective functioning (correlation coefficient of −0.336); and the parent–child relationship is significantly positively correlated with parental reflective functioning (correlation coefficient of 0.625).

**Table 1:** Correlation analysis results of four variables (*N* = 3,166).

Variable	1	2	3	4
1. Social–emotional competence	--			
2. Parenting stress	−0.586**	--		
3. Parent–child relationship	0.659**	−0.644**	--	
4. Parental reflective functioning	0.445**	−0.336**	0.625**	--

*** indicates *p* < 0.001.

### 3.4 Chained mediation effect test

The correlation analysis yielded findings that indicated a strong correlation among the variables, thereby satisfying the fundamental criteria for conducting further mediation effect tests. The mediation effect test was conducted by adopting Model 6 of the PROCESS plug-in SPSS, which was provided by Hayes (2022). The nonparametric bootstrap percentile method was used to sample 5,000 times repeatedly. The parenting stress was used as the independent variable, preschool children’s social–emotional competence as the dependent variable, and the preschool children’s gender, age, whether they were the only child, the location of kindergarten, the parents’ educational background, and the annual household income as control variables.

The results of regression analysis are shown in Table 2: Before adding the mediating variable, a significant direct effect of the parenting stress was found on preschool children’s social–emotional competence. After parental reflective functioning and parent–child relationship were included in the regression equation, a significant negative predictive effect of the parenting stress on parental reflective functioning and parent–child relationship were found; and a significant positive effect of parental reflective functioning and parent–child relationship on preschool children’s social–emotional competence was found. Meanwhile, the parenting stress still exerts a significant negative direct effect on preschool children’s social–emotional competence.

**Table 2:** Summary of chained mediation effect analysis of parental reflective functioning and parent–child relationship.

Title 1	Equation 1		Equation 2		Equation 3		Equation 4	
	Preschool children’s social–emotional competence		Parental reflective functioning		Parent–child relationship		Preschool children’s social–emotional competence	
	*β*	*t*	*β*	*t*	*β*	*t*	*β*	*t*
Parenting stress	−0.338***	−39.793	−0.290***	−18.590	−0.335***	−40.974	−0.165***	−16.847
Parental reflective function	--	--	--	--	0.337***	38.018	0.054***	5.236
Parent–child relationship	--	--	--	--	--	--	0.362***	21.008
The preschool children’s gender	0.078***	4.688	0.068*	2.230	−0.016	−1.075	0.072***	4.871
The preschool children’s age	0.017*	1.966	−0.048**	−3.076	0.001	0.160	0.024**	3.277
Only child or not	0.029	1.537	−0.146***	−4.261	−0.006	−0.336	0.057**	3.407
Location of kindergarten	0.062	3.176	0.110**	3.059	0.043*	2.385	0.027	1.568
Parental education levels	−0.004**	−0.355	0.007	0.335	−0.016	−1.460	0.000	0.024
Annual household income	0.018*	2.182	0.069***	4.645	−0.004	−0.535	0.007	0.962
R^2^	0.353		0.143		0.604		0.492	
F	245.725***		75.490***		601.995***		339.131***	

*p < 0.05, **p < 0.01, ***p < 0.001. Equation 1 represents the direct effect, Equations 2 and 3 represent the paths from the independent variable to the mediating variable, Equation 4 represents the direct effect of the independent variable on the dependent variable, taking into account the mediating variable, as well as the path of the mediating variable to the dependent variable.

**Figure 1:**
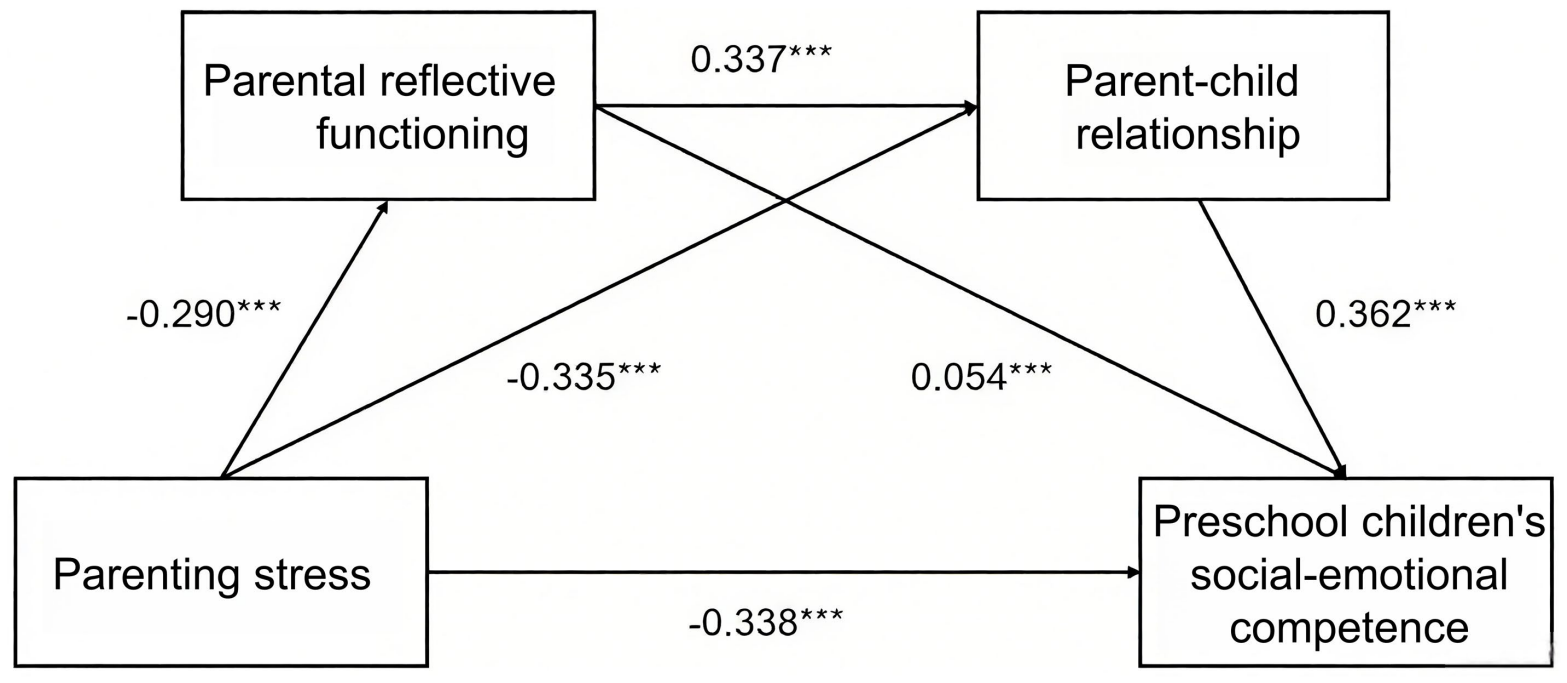
Diagram of chained mediation effect.

The Bootstrap sampling test method was used to test the mediation effect of three paths, sampling 5,000 times. The results are shown in Table 3: In terms of the mediating path of ‘average parenting stress ⇒ average parental reflective functioning ⇒ average social–emotional competence’, the 95% interval does not include zero (95% CI: −0.023 ~ −0.009), proving the existence of this mediating path. In terms of the mediating path of ‘average parenting stress ⇒ average parent–child relationship ⇒ average social–emotional competence’, the 95% interval does not include zero (95% CI: −0.137 ~ −0.106), thus proving the existence of this mediating path. Then, the chained mediating paths were analyzed. In terms of the mediating path of ‘average parenting stress ⇒ average parental reflective functioning ⇒ average parent–child relationship ⇒ average social–emotional competence, the 95% interval does not include zero (95% CI: −0.042 ~ −0.029), proving the existence of this mediating path (the model as shown in Figure 1).

**Table 3:** Summary of mediation effects and confidence intervals.

Mediating path	Effect	Boot SE	Boot LLCI	Boot ULCI	Proportion to the total effect
X-M1-Y	−0.016	0.004	−0.023	−0.009	4.73%
X-M2-Y	−0.122	0.008	−0.137	−0.106	36.09%
X-M1-M2-Y	−0.035	0.003	−0.042	−0.029	10.36%
Total indirect effect value	−0.173	0.009	−0.19	−0.155	51.18%

## 4 Discussion

### 4.1 Relationship between parenting stress and preschool children’s social–emotional competence

Consistent with previous research (Carapito et al., 2018), the results of this study showed that parenting stress is negatively correlated with preschool children’s social–emotional competence, and can be used to significantly predict preschool children’s social–emotional competence. The parenting stress theory points out that great parenting stress will create a tense and unstable family environment, which will influence the children’s emotional security and ability to explore the environment, and finally influence children’s social–emotional development (Wang and Cheng, 2025). Under excessive parenting stress, parents may take more strict and disciplinary actions when disciplining their children, which is more likely to cause behavioral problems in preschool children (Wu et al., 2022) and cause a negative influence on the development of children’s social–emotional competence if they are not properly guided and intervened (Longobardi et al., 2025). According to the parenting stress theory, parents will adopt more negative parenting styles under great parenting stress, including little concern and less positive emotions (Abidin, 1992). Children will observe and imitate their parents’ behaviors to learn social–emotional skills. If parents show negative emotional states because of high stress, children may imitate these emotional states, thus influencing the development of their social–emotional competence. By exploring the relationship between parenting stress and preschool children’s social–emotional competence, we identified the importance of helping parents reduce their parenting stress and the necessity of promoting children’s social–emotional competence development through family intervention and education programs.

### 4.2 The mediating mechanism of parental reflective functioning and the parent–child relationship

Firstly, this study found out that parental reflective functioning can mediate the relationship between parenting stress and preschool children’s social–emotional competence. The results showed that: parents with higher levels of reflective function can think about their psychological states and those of their children, better understand the difficulties in parenting their children and the behaviors of their children, and treat problems arising in the process with an optimistic and positive attitude. Therefore, the reflective function can help parents improve their negative emotions and experiences during parenting and reduce their parenting stress. Meanwhile, as a very important protective factor in parenting adversity (Stuhrmann et al., 2022), the reflective function can guide parents to focus on their children’s psychological state and behaviors. According to mentalization theory, with the accompaniment and education of parents who have secure attachment and mentalization ability, children can understand their emotional state from the outside through mirror reflection, which is conducive to improving their social–emotional competence (Locati et al., 2025). Other studies showed that parental reflective functioning can be used as an important buffer factor in the relationship between parents’ bad emotions/behaviors and children’s development (Dollberg et al., 2021; Shalev et al., 2023). This study again proved that parental reflective functioning can effectively alleviate the negative influence of parenting stress on preschool children’s social–emotional competence.

This study also proved that the parent–child relationship can mediate the relationship between parenting stress and preschool children’s social–emotional competence. Under great parenting stress, parents may show more negative emotions and behaviors, such as irritability, anxiety or depression, which may directly influence their interaction with their children. As a result, parents interact less with their children with decreased positive parent–child interaction and emotional support, which in turn hinders their children from learning social–emotional competence (Wang et al., 2023). According to the spillover hypothesis theory, parents under greater stress are easy to spill their negative experiences to other systems in the family, such as the “parent–child system,” thus influencing their children’s development. In the meantime, the susceptibility-stress-adaptation model shows that greater parenting stress will indirectly influence children’s development through family environment characteristics, e.g., parent–child relationship, husband-wife relationship, etc. (Wang et al., 2020). This model shows the relationship among parenting stress, parent–child relationship and children’s social–emotional competence in a more direct and concrete way. Therefore, this study discusses the influence of parenting stress and parent–child relationship on preschool children’s social–emotional competence, in a bid to provide theoretical reference for family parenting, bring parents’ attention to establishing good parent–child relationships, and improve parent–child relationships.

This study also found out that parenting stress sequentially influences preschool children’s social–emotional competence through the chained mediation of parental reflective functioning and parent–child relationship. The results suggested that when under excessive parenting stress, parents should actively pay attention to their own and their children’s psychological state and behavior, improve their reflective function, change the traditional parent-centered education mode, and build a parent–child relationship with high intimacy and low conflict (Duncan et al., 2009). The harmonious and intimate parent–child relationship is an important external resource for children’s growth, which can promote the formation and development of children’s positive behavior and ability (Coatsworth et al., 2018). The Conservation of Resources emphasizes that when individuals experience positive growth of resources, these new resources will promote each other and form a positive dynamic cycle, which will bring more resources and positive results, thus further enhancing individuals’ adaptability and ability to cope with stress, that is, there will be resource gain spiral effect (Hobfoll et al., 2018). In this study, parents’ strong reflective function and good parent–child relationship are important resources for individual growth. These resources interact with each other to cope with the adverse effects of parenting stress on children’s development, and jointly promote the development of preschool children’s social–emotional competence.

### 4.3 Limitations and suggestions for further research

While our study contributes new insights, it is important to acknowledge several limitations. Firstly, regarding the selection of variables, various factors influence the development of preschool children’s social–emotional competence. This study focuses only on the micro-system (family) of the ecosystem. However, the intermediate, external, and macro systems also influence the development of preschool children in an interacting way. Therefore, future research should comprehensively explore the development of children’s social–emotional competence by considering the various factors that influence it.

Secondly, regarding the selection of the sample area, this study primarily selected children and parents from western China, which limited the distribution of the sample. Therefore, future research should expand the research area to investigate differences in factors influencing preschool children’s social–emotional competence in different regions. This will allow us to describe the characteristics of children’s social–emotional competence development in different regions, as well as the specific effects of parenting stress and reflective function on children’s social–emotional competence development in different regions.

Thirdly, regarding the research methods, all variables in this study were investigated through parental reports. Since participants are likely to evaluate their children according to their own views and expectations, this study can be considered single-source research. Therefore, future research should collect multi-source data from teachers, peers, and children to increase the comprehensiveness and reliability of the data. Regarding the research design, the horizontal design adopted in this study cannot capture dynamic processes that change over time. Therefore, future research should explore the causal relationships among variables through longitudinal follow-up studies.

Finally, this study controlled for demographic variables when examining variable relationships, preliminarily excluding their confounding effects and establishing a foundation for understanding the core relationships of interest. However, the complex interactive relationships between demographic variables themselves and parenting processes, as well as child development, are a topic that merits in-depth investigation. This study provides a starting point and reference for this endeavor. Future research can use this study as a foundation to further elevate demographic factors from “control variables” to “moderators” or “mediators,” exploring how these factors shape the pathways linking parenting stress, parental reflective functioning, and the parent–child relationship to preschool children’s social–emotional competence. This would advance more comprehensive and ecologically valid theoretical models.

## 5 Conclusion

This study focuses on the social–emotional competence of preschool children and explores how parenting stress influences this competence. It proves the independent and chained mediation effects of the parent–child relationship and parental reflective functioning on the relationship between parenting stress and the social–emotional competence of preschool children. This study also provides a theoretical basis and practical support for improving the social–emotional competence of preschool children. Integrating family systems theory, attachment theory, and mentalization theory, the study identifies parents’ internal (reflective functioning) and external (parent–child relationship) developmental resources as internal mechanisms influencing parenting stress and children’s social–emotional competence. The study also verifies the applicability of these theories to preschool children in Western China and provides a theoretical foundation for promoting their healthy development. Practically speaking, this study suggests that parents of pre-school children pay more attention to their children’s development, learn advanced parenting concepts, and practice them in parent–child interactions to improve reflective functioning and promote the harmonious development of the parent–child relationship. This will enhance the development of preschool children’s social–emotional competence.

## Data Availability

The raw data supporting the conclusions of this article will be made available by the authors, without undue reservation.
